# Assessing Tumor Progression Factors by Somatic Gene Transfer into a Mouse Model: Bcl-xL Promotes Islet Tumor Cell Invasion

**DOI:** 10.1371/journal.pbio.0050276

**Published:** 2007-10-16

**Authors:** Yi-Chieh Nancy Du, Brian C Lewis, Douglas Hanahan, Harold Varmus

**Affiliations:** 1 Program in Cancer Biology and Genetics, Memorial Sloan-Kettering Cancer Center, New York, New York, United States of America; 2 Department of Biochemistry, University of California San Francisco, San Francisco, California, United States of America; Washington University School of Medicine, United States of America

## Abstract

Tumors develop through multiple stages, implicating multiple effectors, but the tools to assess how candidate genes contribute to stepwise tumor progression have been limited. We have developed a novel system in which progression of phenotypes in a mouse model of pancreatic islet cell tumorigenesis can be used to measure the effects of genes introduced by cell-type-specific infection with retroviral vectors. In this system, bitransgenic mice, in which the rat insulin promoter (RIP) drives expression of both the SV40 T antigen *(RIP-Tag)* and the receptor for subgroup A avian leukosis virus *(RIP-tva),* are infected with avian viral vectors carrying cDNAs encoding candidate progression factors. Like *RIP-Tag* mice, *RIP-Tag; RIP-tva* bitransgenic mice develop isolated carcinomas by ∼14 wk of age, after progression through well-defined stages that are similar to aspects of human tumor progression, including hyperplasia, angiogenesis, adenoma, and invasive carcinoma. When avian retroviral vectors carrying a green fluorescent protein marker were introduced into *RIP-Tag; RIP-tva* mice by intra-cardiac injection at the hyperplastic or early dysplastic stage of tumorigenesis, approximately 20% of the TVA-positive cells were infected and expressed green fluorescent proteins as measured by flow cytometry. Similar infection with vectors carrying cDNA encoding either of two progression factors, a dominant-negative version of cadherin 1 (dnE-cad) or Bcl-xL, accelerated the formation of islet tumors with invasive properties and pancreatic lymph node metastasis. To begin studying the mechanism by which Bcl-xL, an anti-apoptotic protein, promotes invasion and metastasis, *RIP-Tag; RIP-tva* pancreatic islet tumor cells were infected in vitro with RCASBP-*Bcl-xL*. Although no changes were observed in rates of proliferation or apoptosis, Bcl-xL altered cell morphology, remodeled the actin cytoskeleton, and down-regulated cadherin 1; it also induced cell migration and invasion, as evaluated using two-chamber transwell assays. In addition, myosin Va was identified as a novel Bcl-xL-interacting protein that might mediate the effects of Bcl-xL on tumor cell migration and invasion.

## Author Summary

Cancer cells accumulate multiple genetic alterations. Some of these contribute to tumor development while others are a mere by-product of genomic instability. To determine whether a candidate gene can promote tumor development, we have developed a novel experimental system using engineered viruses to deliver genes into premalignant lesions. We used genetically engineered mice in which both an oncogene (SV40 T antigen) and a specific docking molecule for the virus are produced in β cells in the pancreatic islets of Langerhans. Tumors form in only a subset of the islets expressing this oncogene, indicating that tumor development requires other events. Since these precancerous cells also express the virus docking molecule, we could deliver candidate progression genes via the virus to see whether they contributed to tumor progression. We show that genes encoding two proteins (a survival factor, Bcl-xL, and an inhibitory form of the cell adhesion molecule cadherin 1) can be delivered into premalignant β cells and thereby enhance tumorigenesis. Both of these proteins were previously implicated in tumor progression, confirming that our technique can identify such progression genes. Moreover, we find that Bcl-xL promotes tumor cell migration and invasion by a mechanism distinct from its known role in cell survival.

## Abstract 3

A new technique using lentivirus to deliver genes into targeted cell types reveals that the tumor progression gene*Bcl-xL* not only promotes cell survival in premalignant cells but also promotes tumor cell invasion.

## Introduction

Cancer typically arises by a multi-step process during which cells acquire activating mutations in oncogenes and inactivating mutations in tumor suppressor genes [1]. By comparing cancer tissues with their normal counterparts, it is possible to identify many differences in cellular, molecular, and biological properties. For example, transcriptional profiling and genome sequencing methods have documented alterations in gene expression and somatic mutations in cancers [2,3]. Several types of cancers share common somatic changes or display changes in the same functional pathways, revealing the underlying principles that drive the transformation of normal cells into highly malignant derivatives [4].

Mouse models of human cancers provide important experimental systems for understanding the complexities of human cancer pathogenesis [5,6]. However, in most mouse models, mutant genes have been more clearly implicated in the initiation than in the progression of cancer, even when newer methods, such as Cre/*loxP*-mediated recombination or tetracycline- or estrogen-based gene regulation, are used to produce a particular gene product conditionally in a tissue-specific and time-controlled manner [7,8]. Moreover, it is time-consuming and expensive to generate alterations in the mouse germ line for each gene of interest.

Seeking to improve means for studying later stages in tumorigenesis, we have developed a strategy for assessing causal links between individual genes and specific cellular changes during tumor progression. The strategy is based on the use of a transgene that encodes the receptor for subgroup A avian leukosis virus, TVA, allowing cell-specific infection with avian retroviral vectors during tumor development [9,10]. The *RIP1-Tag2* (or *RIP-Tag*) transgenic mouse [11] was chosen as a platform to study the effects of candidate genes in tumor progression. In this well-characterized model, SV40 T antigen is expressed under the control of the Rat insulin promoter (RIP) in the β cells of pancreatic islets, providing the driving force for tumor initiation by blocking the activities of the Rb and p53 tumor suppressors. The transgenic mice develop multifocal pancreatic β-cell tumors through well-defined stages that resemble lesions of human tumor development and progression. The natural focal distribution of the target tissue, into ∼400 focal nodules, has facilitated identification and quantification of the distinguishable stages in islet tumorigenesis, which include hyperplasia or low-grade dysplasias (“hyperplastic islets”), high-grade angiogenic dysplasias (“angiogenic islets”), encapsulated, noninvasive islet tumors (IT, adenomas), and distinctive grades of invasive carcinoma [12].

Using a *RIP-tva* transgene to express the TVA receptor in β cells of the pancreas, we demonstrate that candidate genes can be introduced somatically into developing neoplastic β- cell lesions in *RIP-Tag; RIP-tva* bitransgenic mice by infection with a subgroup A avian retroviral vector, termed RCASBP (subgroup A replication-competent avian leukosis virus with a splice acceptor and the Bryan-RSV *pol* gene) [13,14], thereby modulating tumorigenesis. By doing so, we have established the functional roles of two candidate progression factors, a dominant-negative form of cadherin 1 (dnE-cad) and Bcl-xL, when introduced stochastically in incipient neoplasias. Our study also reveals an unexpected activity of Bcl-xL in altering the actin cytoskeleton and promoting invasiveness, findings substantiated by analysis of cultured tumor cells. Our results suggest that targeted somatic delivery of candidate progression factors via retrovirus vectors will be useful for identifying factors and dissecting mechanisms that underlie advanced stages of tumorigenesis.

## Results

### Generation and Characterization of *RIP-tva* Transgenic Mice

**Figure 1:**
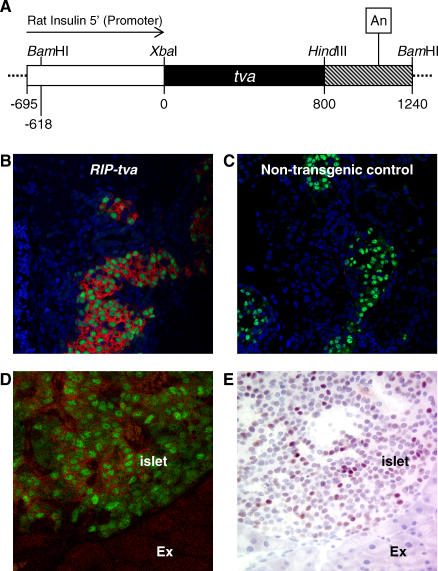
Characterization of *RIP-tva* Transgenic Mice (A) The *RIP-tva* transgene. The 800-bp *tva* cDNA was cloned into a XbaI/HindIII-digested plasmid that contains the 5′ flanking region of the rat insulin II gene and transcription termination sequences (An) (details in Materials and Methods). (B and C) Confocal microscopic images show immunofluorescent membrane staining of TVA (red) and nuclear staining of PDX1 (green) in pancreatic sections of 2-d-old *RIP-tva* transgenic (B) and non-transgenic (C) animals. Nuclei were stained with DAPI (blue). Original magnification, 630×. The images are representative of 20 fields from 12 mice analyzed. (D) Confocal microscopic immunofluorescent image of hyperplastic islet cells revealed TVA (red) and PDX1 (green) in pancreatic section of a 7-wk-old *RIP-Tag; RIP-tva* bitransgenic animal. The image is representative of 15 fields from three mice analyzed. (E) The adjacent section of (D) was immunostained for SV40 T antigen. The image is representative of 15 fields from three mice analyzed. Ex, exocrine tissue.

The general strategy employed in this work involves equipping pancreatic β cells with TVA, the cell surface receptor for subgroup A avian leukosis viruses [9,10], so that the cells are susceptible to the RCASBP vector [15] derived from those viruses. To that end, we generated transgenic mice that express TVA under the control of the RIP exclusively in the β cells of pancreatic islets (Figure 1A–1C). Three independent lines of *RIP-tva* mice transmitted the transgene in a simple Mendelian pattern of inheritance.

To detect TVA on the surface of pancreatic cells in *RIP-tva* newborn and adult mice, we performed immunofluorescent staining with a rabbit polyclonal anti-TVA antibody on pancreatic sections. To identify pancreatic islets, we co-stained the sections with goat antisera against PDX1, a transcription factor expressed in pancreatic precursor cells and islet β cells. In both newborn and adult animals, TVA was observed in cells within the islets in all three *RIP-tva* transgenic lines with similar expression levels (Figure 1B and data not shown). No TVA staining was detected in either exocrine cells from the *RIP-tva* mice (Figure 1B) or pancreases from non-transgenic animals (Figure 1C). In addition, expression of TVA in other organs was not detected by immunofluorescent staining (data not shown). We conclude that TVA is produced specifically in the pancreatic β islets of *RIP-tva* transgenic mice, although we cannot rule out the possibility that other cell types may express the transgene at levels below those detectable with the method employed.

Examination of pancreatic tissues from newborn and adult animals did not reveal any significant differences in islet morphology between *RIP-tva* animals and non-transgenic littermates (data not shown). The distribution and presence of cells expressing insulin II (known as insulin), glucagon, somatostatin, and pancreatic polypeptide were also similar in *RIP-tva* mice and non-transgenic littermates (data not shown), indicating that the viral receptor, TVA, itself did not perturb any of the properties of islet cells that might be relevant to this study.

### Gene Transfer to *RIP-Tag; RIP-tva* Cells *In Vitro* and *In Vivo*

**Figure 2:**
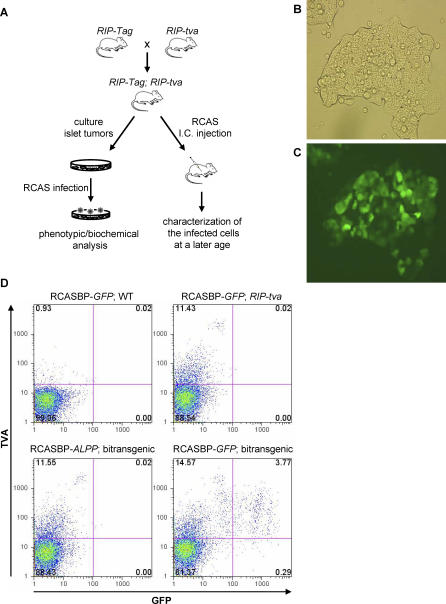
Infection of *RIP-Tag; RIP-tva* Pancreatic Islet Cells In Vitro and In Vivo (A) Experimental strategy. *RIP-Tag* transgenic mice were crossed with *RIP-tva* transgenic mice to generate *RIP-Tag; RIP-tva* bitransgenic mice. For in vitro experiments, tumors from 16-wk-old bitransgenic mice were isolated and cultured in vitro, and tumor cells were infected with RCASBP viruses (left). For in vivo infection, RCASBP viruses were delivered into 7-wk-old bitransgenic animals through intra-cardiac (I.C.) injection. Characterization of islet cells/tumors was performed 2, 5, or 9 wk after viral delivery (right). (B and C) Example of infection in vitro with an RCASBP vector. Bright-field image of *RIP-Tag; RIP-tva* tumor cells infected with RCASBP*-GFP* in vitro (B) and fluorescent image showing GFP expression (C) were taken 1 wk after infection. The images are representative of more than 20 fields from two independent infections. (D) Flow cytometry plots of islet cells from wide-type mouse (C57BL/6) injected with RCASBP*-GFP* (upper left), *RIP-tva* transgenic mouse injected with RCASBP*-GFP* (upper right), and *RIP-Tag; RIP-tva* bitransgenic mice injected with RCASBP*-ALPP* (lower left) or RCASBP*-GFP* (lower right) via intra-cardiac injection. RCASBP viral supernatants were delivered at the age of 7 wk. Two weeks later, mice were sacrificed, and pancreases were digested into single-cell suspension. Cells were analyzed for TVA^+^ and GFP by FACS. The percentage of cells in each gated panel is indicated in the corners. Approximately 11%–18% of pancreatic cells isolated from *RIP-tva* or *RIP-Tag; RIP-tva* bitransgenic mice by this method were TVA^+^ (upper quadrants), and about 20% of TVA^+^ cells from bitransgenic animals infected with RCASBP*-GFP* expressed GFP.

To prepare to study the effects of candidate progression factors, introduced by virus infection, on the course of tumor development, one of the three *RIP-tva* lines was crossed with the *RIP-Tag* line to generate bitransgenic *RIP-Tag; RIP-tva* mice (Figure 2A). It has been shown that the strain backgrounds influence the tumorigenesis of *RIP-Tag* mice, and the course of tumorigenesis is more consistent in a C57BL/6 background [16,17]. We therefore backcrossed *RIP-tva* mice from a mixed genetic background for ten generations onto a pure C57BL/6 background with *RIP-Tag*.

We initially verified the co-production of TVA and SV40 T antigen in the islets of 7-wk-old bitransgenic mice (Figure 1D and 1E). We then determined whether the *RIP-tva* transgene conferred susceptibility to infection by RCASBP viruses in islet cells derived from the bitransgenic mice. Cell proliferation is required for successful infection by RCASBP retroviruses, but adult islet cells have low proliferation rates [18]. To increase the fraction of proliferating cells, cultures were established from β-cell tumors from *RIP-Tag; RIP-tva* bitransgenic mice, and the tumor cells were infected with a RCASBP vector carrying the green fluorescent protein (GFP) marker (RCASBP*-GFP*). Analysis by fluorescence microscopy at 1 wk after the second of two rounds of infection revealed that more than 70% of the tumor cells expressed GFP, demonstrating that cells derived from the β-cell tumors were readily infectable by RCASBP in vitro (Figure 2B and 2C). No green fluorescence was detectable in uninfected cells (data not shown).

We next evaluated gene transfer in vivo. We chose to infect *RIP-Tag; RIP-tva* bitransgenic animals at 7 wk after birth, an age when hyperplastic islets develop in *RIP-Tag* mice. A concentrated preparation of RCASBP*-GFP* viruses (0.1 ml; >10^8^ infectious units per milliliter) was delivered by intra-cardiac injection to ensure distribution of viruses to all organs fed by the arterial circulation. Two weeks after infection, the percentage of GFP-expressing TVA-positive cells in the islets was determined by fluorescence-activated cell sorting (FACS) (Figure 2D). Suspensions of single pancreatic cells were prepared as described in Materials and Methods, using a protocol modified from Shih et al. [19] and Dor et al. [20]. Approximately 11%–18% of the cells in these preparations were TVA-positive when isolated from bitransgenic animals, and about 20% of the TVA-positive cells expressed GFP (Figure 2D, lower right panel), implying that the infection efficiency in vivo is around 20% in TVA-positive cells. The green fluorescence was not due to autofluorescence, because almost no fluorescence was observed in pancreatic cells from wild-type or *RIP-tva* monotransgenic animals injected with RCASBP*-GFP* or from bitransgenic mice injected with RCASBP viruses encoding human placental alkaline phosphatase (RCASBP-*ALPP;* previously known as RCASBP*-AP*) (Figure 2D). The lack of detectable infection in adult *RIP-tva* monotransgenic animals injected with RCASBP*-GFP* confirmed that proliferation of the TVA-expressing cells is necessary for successful infection.

### dnE-cad Promotes Invasive Tumors and Metastasis in *RIP-Tag; RIP-tva* Mice

To ascertain whether the development of β-cell tumours could be accelerated by the somatic delivery of factors previously reported to promote tumor progression in *RIP-Tag* mice, we infected *RIP-Tag; RIP-tva* bitransgenic mice with RCASBP vectors that carry cDNA encoding dnE-cad, a mutant protein lacking the extracellular, adhesion-mediating domain of cadherin 1 [21]. In previous studies involving *RIP1-Tag2; RIP1-dnE-cad* bitransgenic mice, suppression of cadherin 1 function by expression of a *dnE-cad* cDNA enhanced and accelerated the progression from noninvasive ITs (adenomas) to invasive carcinomas and increased the frequency of otherwise rare lymph node metastasis [22].

**Figure 3:**
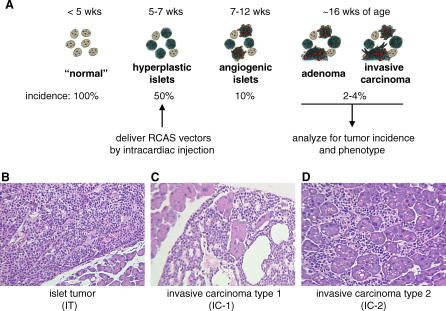
*RIP-Tag; RIP-tva* Islet Tumorigenesis and Examples of Histological Categories (A) Schematic representation of the *RIP-Tag; RIP-tva* islet tumorigenesis (modified from the *RIP1-Tag2* Manual, Hanahan laboratory, unpublished). RCASBP retroviruses carrying genes of interest were introduced into the hyperplastic islets of 7-wk-old mice through intra-cardiac injection. Mice were sacrificed at 16 wk of age for histological staging and grading to determine tumor incidence and phenotype as shown in Table 1. (B–D) Hematoxylin and eosin stain of representative examples of IT (B), IC-1 (C), and IC-2 (D).

We injected high titer stocks (>10^8^ infectious units per milliliter) of RCASBP viruses carrying cDNA encoding dnE-cad into 7-wk-old *RIP-Tag; RIP-tva* bitransgenic animals by the intra-cardiac route (Figure 3A). Nine weeks later, pancreases from 16-wk-old mice were harvested for histological staging and grading of the lesions according to established criteria [23]. In brief, tumors were scored as IT (noninvasive tumor; >1 mm in diameter; Figure 3B); invasive carcinoma type 1 (IC-1; >1 mm in diameter; focal regions of invasion; Figure 3C); or invasive carcinoma type 2 (IC-2; variable sizes; widespread invasion; Figure 3D). Pancreatic sections were also subjected to immunohistochemical tests for the expression of synaptophysin, a neuroendocrine marker found in all types of islet cells, and for insulin II, a β-cell marker.

**Table 1:**
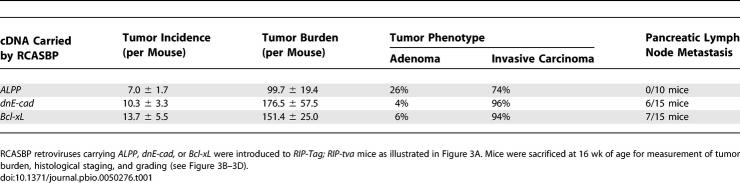
Impact of RCAS*-Bcl-xL* and RCAS*-dnE-cad* on Tumorigenesis In Vivo

**Figure 4:**
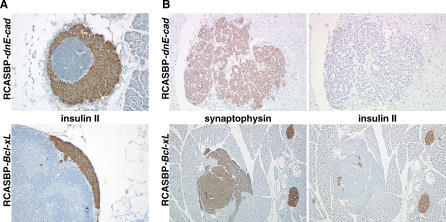
Detection of Metastasis and Assessment of the Differentiation Status of IC-2 from Mice Infected with RCASBP*-Bcl-xL* and RCASBP*-dnE-cad* (A) Representative images of lymph node metastases found in six mice infected with RCASBP*-dnE-cad* (upper panel) or in seven mice infected with RCASBP*-Bcl-xL* (lower panel). Immunohistochemical staining of insulin II in metastatic β-cell tumor cells in pancreatic lymph nodes is shown. Original magnification, 200×. (B) About 4.5% of IC-2 found in mice infected with RCASBP*-dnE-cad* or RCASBP*-Bcl-xL* do not express insulin II. Immunohistochemical staining of synaptophysin, a neuroendocrine marker (left), and insulin II (right) in β-cell tumor cells in pancreatic sections from mice injected with RCASBP*-dnE-cad* (upper panel) or RCASBP*-Bcl-xL* (lower panel). Original magnification, 200× (upper panel) and 100× (lower panel).

By counting and classifying pancreatic lesions (Table 1), we found that (i) the average tumor incidence in bitransgenic mice infected with RCASBP*-dnE-cad* (*n =* 11) was 1.5-fold higher than that in bitransgenic mice injected with the control RCASBP*-ALPP* viruses (*n =* 6); (ii) mice injected with RCASBP*-dnE-cad* (*n =* 11) exhibited a higher incidence of invasive carcinomas than mice injected with the control viruses (*n =* 6); and (iii) mice injected with RCASBP*-dnE-cad* had a higher tumor burden than controls (*p* = 0.003, *n =* 6 for each group, Wilcoxon rank sum test). By staining pancreatic lymph nodes with antisera against insulin II and synaptophysin to search for metastatic β-cell tumors, we found that six of the 15 mice infected with RCASBP*-dnE-cad* developed metastases to the pancreatic lymph nodes (Table 1; Figure 4A, upper panel); metastases were not detected in monotransgenic *RIP-Tag* mice of the same age or in *RIP-Tag; RIP-tva* bitransgenic mice infected with RCASBP*-ALPP*. No metastases to other distant organs (liver, kidney, lung, heart, or thymus) were observed in the animals infected with RCASBP*-dnE-cad* or RCASBP*-ALPP,* consistent with the findings in *RIP1-Tag2; RIP1-dnE-cad* bitransgenic mice. Taken together, these data indicate that the *RIP-Tag; RIP-tva* mice can be used to assess the effects of genes of interest on tumor progression in vivo, with targeted somatic delivery of *dnE-cad,* largely recapitulating the effects seen with a traditional transgenic approach that constitutively expresses the candidate progression factor in all of the target cells throughout ontogeny [22].

### Bcl-xL Increases Tumor Invasion and Metastasis in *RIP-Tag; RIP-tva* Mice

We next investigated a second progression factor, Bcl-xL, a member of the anti-apoptotic subgroup of the BCL2 family of apoptotic regulators [24] via somatic gene transfer. In previous studies, constitutive expression of *Bcl-xL* via a *RIP7-Bcl-xL* transgene was found to suppress apoptosis and alternatively accelerate or enable islet tumorigenesis in mouse models of cancer evoked by SV40 T antigen [25] or MYC [26], respectively. We sought to assess the effects of expressing exogenous *Bcl-xL* in sporadic cells, beginning after neoplastic development had ensued, as contrasted to its constitutive expression throughout tumorigenesis in the bitransgenic *RIP1-Tag2; RIP7-Bcl-xL* mice.

We injected high titer stocks of RCASBP viruses carrying cDNA encoding Bcl-xL into 7-wk-old *RIP-Tag; RIP-tva* bitransgenic mice, and subsequently harvested pancreases at 16 wk of age for histopathology (Table 1). We observed that (i) the average tumor incidence in bitransgenic mice infected with RCASBP*-Bcl-xL* (*n =* 10) was 2-fold higher than that in bitransgenic mice injected with the control RCASBP*-ALPP* viruses (*n =* 6); (ii) mice infected with RCASBP*-Bcl-xL* (*n =* 10) exhibited a higher incidence of invasive carcinomas than did mice that received the control viruses (*n =* 6); and (iii) mice infected with RCASBP*-Bcl-xL* displayed a higher tumor burden than controls (*p* = 0.003, *n =* 6 for each group, Wilcoxon rank sum test). We also found that seven of the 15 mice infected with RCASBP*-Bcl-xL* developed metastases to the pancreatic lymph nodes, but no metastases to other organs (Table 1; Figure 4A, lower panel). Therefore, overexpression of *Bcl-xL*, beginning in hyperplastic or early dysplastic lesions, after tumorigenesis had been initiated, increased tumor burden, incidence, and invasion, and facilitated lymph node metastasis (*p* = 0.05, Fisher's exact test).

Histological analysis of the tumors revealed that insulin II expression was not detectable in a small subset (∼4.5%) of IC-2 in *RIP-Tag; RIP-tva* mice infected with RCASBP*-Bcl-xL* or RCASBP*-dnE-cad* (Figure 4B, right panels). All tumors stained with antisera recognizing the neuroendocrine marker synaptophysin (Figure 4B, left panels), suggesting a process of de-differentiation or transformation of less mature cells into an “anaplastic” islet carcinoma, a phenotype not observed in bitransgenic animals that were uninfected or infected with control vectors.

**Figure 5:**
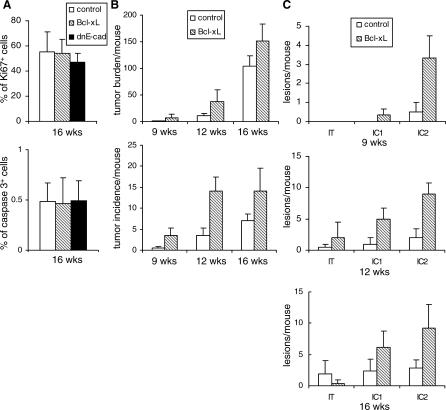
RCASBP*-Bcl-xL* Enhances Tumorigenesis in *RIP-Tag; RIP-tva* Mice *RIP-Tag; RIP-tva* mice were injected with RCASBP*-ALPP* (control), RCASBP*-Bcl-xL,* or RCASBP*-dnE-cad* at 7 wk of age, and were sacrificed at the indicated ages for histopathological analysis. (A) Proliferative index (upper panel) and apoptotic index (lower panel) in the tumors from 16-wk-old mice injected with RCASBP*-ALPP* (control), RCASBP*-Bcl-xL,* or RCASBP*-dnE-cad* were determined. Pancreatic sections were stained with antibodies against Ki67 or activated caspase 3. Data shown are the mean percentage ± standard deviation from ten tumors of each group. (B and C) Mice injected with RCASBP*-ALPP* (control) or RCASBP*-Bcl-xL* were sacrificed at the indicated ages to evaluate tumor burden (B, upper panel). A standard formula for tumor volume was applied (volume [mm^3^] = 0.52 × width^2^ × length). Tumor burden per mouse is the sum of the tumor volume. Pancreatic sections were stained with synaptophysin for identification of islet cells. Tumor incidence was determined (B, lower panel), and the different categories of tumors were counted (C).

Bcl-xL is known to protect cells from apoptosis [24]. We therefore investigated whether the increased invasive and metastatic behavior of the tumors from mice infected with RCASBP*-Bcl-xL* was due to increased cell survival, producing greater cell numbers and a higher probability of tumor spread, or due to some other properties of Bcl-xL. We observed no significant differences in proliferative or apoptotic indices by staining tumors from 16-wk-old mice previously infected with RCASBP*-ALPP,* RCASBP*-Bcl-xL,* or RCASBP*-dnE-cad* with antisera against a proliferation marker, MKI67 (antigen identified by monoclonal antibody Ki67), or against activated caspase 3 (Kruskal-Wallis nonparametric test, three-way test, *p* = 0.79 and 0.69, respectively) (Figure 5A). These results suggest that the increased invasive and metastatic properties were not due to the effects of Bcl-xL on proliferation or survival of the cancer cells at the stages of adenomas and carcinomas. However, it remains possible that Bcl-xL provides some protection from apoptosis at the metastatic stage or earlier stages in tumorigenesis, as cell death is greatly reduced in both hyperplastic and angiogenic islets in *RIP1-Tag2; RIP7-Bcl-xL* bitransgenic mice that constitutively express high levels of Bcl-xL in all β cells in normal islets and at all stages of tumorigenesis [25]. Since only a small fraction of the β cells in premalignant lesions became somatically infected in our experiments, such effects would likely be masked by the preponderance of uninfected cells in those lesions.

To determine whether the effects of Bcl-xL occurred early or late in the neoplastic process, we examined sections of pancreases from *RIP-Tag; RIP-tva* mice at 2 and 5 wk after RCASBP*-Bcl-xL* delivery (9 and 12 wk of age, respectively), as well as 9 wk after infection (Figure 5B and 5C). This experiment not only confirmed the findings presented in Table 1 at late times after infection, but also showed that the effects of Bcl-xL could be observed as early as 2 wk after viral delivery. *RIP-Tag; RIP-tva* mice infected with RCASBP*-Bcl-xL* exhibited higher tumor burdens (for three ages *p* ≤ 0.009, Wilcoxon rank sum test) and a greater tumor incidence at all three ages (9, 12, and 16 wk of age) than did the bitransgenic control mice infected with RCASBP*-ALPP* (Figure 5B). In addition, at all ages the numbers of IC-1 and IC-2 were higher in mice infected with RCASBP*-Bcl-xL* than in mice that received RCASBP*-ALPP;* furthermore, the small invasive carcinomas appeared before ITs in *RIP-Tag; RIP-tva* mice at 2 wk after RCASBP*-Bcl-xL* delivery (Figure 5C). These findings suggest that some of the hyperplastic or angiogenic islets develop highly invasive carcinomas (variable sizes) without progressing through a noninvasive IT stage (>1 mm in diameter), a phenotype first observed in bitransgenic mice that express both SV40 T antigen and a receptor tyrosine kinase, the insulin-like growth factor (IGF) 1 receptor (IGF1R), in the pancreatic islets [23]. No significant effects of Bcl-xL on proliferative or apoptotic indices in the tumors were observed at 2 and 5 wk after viral delivery (data not shown). Thus, introducing RCASBP*-Bcl-xL* into *RIP-Tag; RIP-tva* mice with islet cell hyperplasia has a substantial impact on tumor burden, tumor incidence, and invasive potential within 2 wk after infection.

### Confirmation of the Presence of RCASBP*-Bcl-xL* Proviral DNA in Tumors and Metastases from Mice Infected with RCASBP*-Bcl-xL*

**Figure 6:**
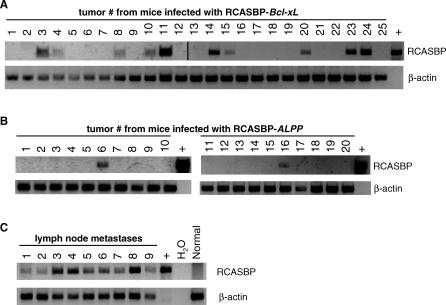
PCR Analysis to Verify the Presence of the RCASBP Proviral DNA in the Tumors (A and B) Tumor DNAs were extracted from freshly microdissected tumors or paraffin-embedded pancreatic sections from mice 9 wk after mice were infected with RCASBP*-Bcl-xL* or RCASBP*-ALPP*. PCR was performed using RCASBP-specific primers or ACTB (β-actin)–specific primers. The presence of RCASBP proviruses in the genomic DNA was detected in ten of 25 tumors from mice infected with RCASBP*-Bcl-xL* (A) and two of the 20 tumors from mice infected with RCASBP*-ALPP* (B). (C) The presence of RCASBP proviruses in the genomic DNA was detected in nine of nine pancreatic lymph node metastases from mice infected with RCASBP*-Bcl-xL* (*n =* 4) or RCASBP*-dnE-cad* (*n =* 5). +, RCASBP plasmid DNA used as template DNA in PCR; Normal, normal lymph node.

Since infection of bitransgenic mice with RCASBP*-Bcl-xL* increases the number of tumors by about 2-fold at 16 wk of age (Table 1; Figure 5), we anticipated that about half of the tumors would contain avian proviral DNA as evidence of infection that confers a selective growth advantage. To test this prediction, we isolated genomic DNA from freshly microdissected tumors or from tumors in paraffin-embedded sections, and we then performed PCR using primers specific to RCASBP sequences. Using materials from the experiment shown in Figure 5, we detected RCASBP DNA in eight of 11 tumors (72%), six of 12 tumors (50%), and ten of 25 tumors (40%) harvested 2, 5, and 9 wk after viral delivery, respectively (Figure 6A and data not shown). In contrast, RCASBP DNA was detectable in only two of 20 tumors from mice sacrificed 9 wk after infection with the control RCASBP*-ALPP* viruses (Figure 6B), which is consistent with an in vivo infection efficiency of at least 10%. These results support the hypothesis that infection leading to production of Bcl-xL, but not the control infection with RCASBP*-ALPP,* provides a selective growth advantage to islet cells during tumorigenesis in *RIP-Tag; RIP-tva* mice.

Because only small amounts of DNA were obtained from these small tumors, we were unable to assess the clonality of tumors containing RCASBP*-Bcl-xL* by restriction mapping and thereby determine if they arose from the expansion of single infected cells. In addition, no suitable anti-Bcl-xL antibodies were found to identify tumors expressing Bcl-xL by immunohistochemistry. We observed lymph node metastases only in mice infected with RCASBP*-Bcl-xL* or RCASBP*-dnE-cad*, not in mice infected with RCASBP*-ALPP,* so we considered the possibility that metastases had arose from tumor cells expressing Bcl-xL or dnE-cad. We confirmed the presence of RCASBP proviral DNA in all nine lymph node metastases from mice infected with RCASBP*-Bcl-xL* or RCASBP*-dnE-cad* (Figure 6C). We cannot, however, claim that all the metastatic cells were derived from RCASBPP*-Bcl-xL-* or RCASBP*-dnE-cad*-infected cells.

### Bcl-xL Induces Changes in Cell Morphology, Migration, and Invasion In Vitro

**Figure 7:**
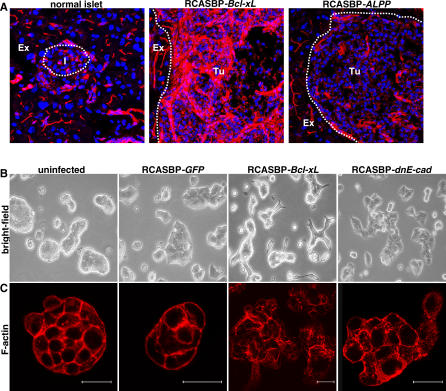
Redistribution of the Actin Cytoskeleton in RCASBP*-Bcl-xL*-Infected Tumor Cells (A) Frozen pancreatic sections from a wild-type mouse (left panel), a mouse 2 wk after RCASBP*-Bcl-xL* infection (middle panel), and a mouse 5 wk after RCASBP*-ALPP* infection (right panel) were stained with rhodamine-phalloidin for F-actin (red) and DAPI (blue). The presence of RCASBP*-Bcl-xL* proviral DNA in the tumor (middle panel) was confirmed by PCR. Three mice each at 2 and 5 wk after RCASBP*-Bcl-xL* delivery and at 5 wk after RCASBP*-ALPP* delivery were analyzed. Ex, exocrine tissue; I, islet; Tu, tumor tissue. (B and C) *RIP-Tag; RIP-tva* tumor cells change morphology in vitro after being infected with RCASBP*-Bcl-xL*. (B) Bright-field images of uninfected tumor cells and cells infected with RCASBP*-GFP,* RCASBP*-Bcl-xL,* or RCASBP*-dnE-cad*. Tumor cells infected with RCASBP*-Bcl-xL* have a less organized epithelial sheet structure. Original magnification, 200×. (C) Tumor cells were cultured on chamber slides and immunostained with rhodamine-phalloidin for F-actin. Tumor cells infected with RCASBP*-Bcl-xL* have decreased cortical actin. Scale bars, 20 μm. The images are representative of more than ten fields in each experiment.

Since tumor invasion and metastasis often involve modulation of the actin cytoskeleton to increase cell motility [27,28], we evaluated the actin cytoskeleton in tumors from *RIP-Tag; RIP-tva* mice infected with RCASBP*-Bcl-xL,* using an immunofluorescence assay with rhodamine-phalloidin to display filamentous actin (F-actin). We observed a dramatic rearrangement of the actin cytoskeleton in invasive carcinomas at 2 and 5 wk after infection with RCASBP*-Bcl-xL,* but not in tumors from age-matched animals infected with RCASBP*-ALPP* (Figure 7A), suggesting that Bcl-xL remodels the actin cytoskeleton to increase cell motility.

To further understand the effects of Bcl-xL on tumor cells, we infected a β-cell tumor cell line (βTC-N134) derived from an uninfected *RIP-Tag; RIP-tva* mouse with RCASBP*-Bcl-xL*. In addition, we infected the tumor cells with RCASBP*-dnE-cad* or RCASBP*-GFP,* to control for any effects of retroviral infection. Two weeks after infection with RCASBP*-Bcl-xL,* the tumor cells became more elongated compared to the uninfected parental cells and to the cells infected with either RCASBP*-GFP* or RCASBP*-dnE-cad* (Figure 7B). Staining with rhodamine-phalloidin demonstrated decreased cortical actin and a less organized epithelial sheet in tumor cells infected with RCASBP*-Bcl-xL,* but not in the other cultures (Figure 7C). Taken together, these results suggest that Bcl-xL promotes remodeling of the actin cytoskeleton, affecting cell shape and adhesion.

**Figure 8:**
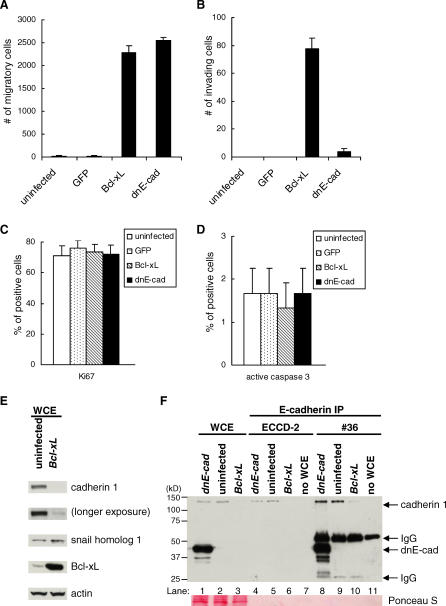
Bcl-xL Has Profound Effects on Cell Migration and Invasion, and Reduces Cadherin 1 In Vitro (A and B) Cell migration and invasion. Uninfected tumor cells or tumor cells infected with RCASBP*-GFP,* RCASBP*-Bcl-xL,* or RCASBP*-dnE-cad* were plated in the upper chambers of transwell plates (A) or of Matrigel invasion plates (B). Data are presented as the mean numbers of cells counted in the lower chambers in five fields under 20× magnification after 72 h, and are representative of three independent experiments. (C and D) Proliferative and apoptotic indices of the four different cell lines. Cultures grown on chamber slides from uninfected tumor cells or cells infected with RCASBP*-GFP,* RCASBP*-Bcl-xL,* or RCASBP*-dnE-cad* were stained with antisera against Ki67 (C) or activated caspase 3 (D). Data shown are the mean percentage ± standard deviation from triplicate experiments. (E) Reduced protein levels of cadherin 1 and elevated snail homolog 1 levels in the tumor cell lines infected with RCASBP*-Bcl-xL*. Whole cell extracts (WCE; 20 μg) from uninfected tumor cells or tumor cells infected with RCASBP*-Bcl-xL* were analyzed by Western blotting using antisera against the cytosolic domain of cadherin 1 (clone 36), snail homolog 1, and Bcl-xL. Actin was measured as a loading control. (F) Two anti–cadherin 1 antibodies (clones ECCD-2 and 36) were used to perform IP from whole cell extracts of uninfected tumor cells and tumor cells infected with RCASBP*-dnE-cad* or RCASBP*-Bcl-xL*. The precipitates were subjected to Western blotting using an antibody against the cytosolic domain of cadherin 1 (clone 36). Two lanes without WCE were used to identify the bands attributable to IgGs (lanes 7 and 11). A portion of nitrocellulose membrane stained with Ponceau S is shown as a loading control.

To examine whether Bcl-xL increased the ability of morphologically altered cells to migrate or invade, we performed two-chamber migration and invasion assays. In the migration assay, uninfected tumor cells (βTC-N134) or tumor cells infected with RCASBP*-GFP,* RCASBP*-Bcl-xL,* or RCASBP*-dnE-cad* were seeded in the upper chambers of transwell inserts with 8-μm porous polycarbonate membranes. We then measured cell migration along a serum gradient through the membrane to the bottom of the chambers. After a 72-h incubation, the number of migratory tumor cells infected with RCASBP*-Bcl-xL* or RCASBP*-dnE-cad* was approximately 1,000-fold higher than that of uninfected cells or cells infected with RCASBP*-GFP* (Figure 8A). In the invasion assay, 8-μm porous polycarbonate membranes were replaced with polyester membranes coated with basement membrane matrix, Matrigel. The tumor cells infected with RCASBP*-Bcl-xL* invaded through Matrigel about 20-fold more efficiently than those infected with RCASBP*-dnE-cad,* and none of the uninfected parental cells or the cells infected with RCASBP*-GFP* had the ability to invade (Figure 8B). The results reveal that Bcl-xL has profound effects on cell migration and invasion in vitro.

To ensure that any increase in cell migration and invasion in vitro was not simply due to an increase of cell number by the anti-apoptotic effect of Bcl-xL, immunocytochemistry using antibodies for Ki67 and activated caspase 3 was performed to assay cell proliferation and cell survival. The frequency of Ki67-positive cells and activated-caspase-3-positive cells remained similar in all three of the infected tumor cell lines compared to the uninfected parental cell line (Figure 8C and 8D). These results, consistent with our in vivo data (Figure 5A), suggest that Bcl-xL is able to promote cell migration and invasion without changing cell proliferation or survival.

### Reduction of Endogenous Cadherin 1 in Tumor Cells Infected with RCASBP*-Bcl-xL*

In addition to the cytoskeleton rearrangement, loss of cadherin 1 can also confer invasive properties on tumor cells [22]. To examine if the expression of cadherin 1 was altered in the tumor cells infected with RCASBP*-Bcl-xL,* we measured cadherin 1 levels by Western blot analysis using an anti–cadherin 1 antibody (clone 36) (Figure 8E). Cadherin 1 protein was much less abundant in the tumor cells infected with RCASBP*-Bcl-xL,* suggesting that overexpression of *Bcl-xL* down-regulates *cadherin 1*. We also observed moderately increased amounts of snail homolog 1 protein (Figure 8E), a transcriptional repressor of cadherin 1 [29,30]. We also performed immunoprecipitation (IP) experiments using two anti–cadherin 1 antibodies (clones ECCD-2 and 36) and whole cell extracts prepared from the uninfected tumor cells or from the tumor cells infected with RCASBP*-dnE-cad* or RCASBP*-Bcl-xL*. The immuoprecipitates were subjected to Western blotting using the clone 36 antibody, which was raised against the cytoplasmic domain of human cadherin 1 and cross-reacts with the mouse homolog. ECCD-2 was raised against a mouse liver cadherin 1 fragment [31], and it did not recognize dnE-cad (Figure 8F, lane 4). The levels of endogenous cadherin 1 were not affected by overexpression of dnE-cad in the tumor cells infected with RCASBP*-dnE-cad* (Figure 8F, lane 1). In marked contrast, cadherin 1 was considerably reduced in the tumor cells infected with RCASBP*-Bcl-xL* (Figure 8F, lanes 3, 6, and 10).

To determine whether regulation of cadherin 1 levels was a result of lowered mRNA levels, we surveyed tumor cell RNAs with oligonucleotide arrays (Affymetrix). We observed that the levels of cadherin 1 RNA were 36-fold lower in *RIP-Tag; RIP-tva* tumor cells infected with RCASBP*-Bcl-xL* than in uninfected parental cells (data not shown), suggesting that Bcl-xL is likely to lower expression of *cadherin 1* at the transcriptional level. Surprisingly, we did not detect a significant reduction of cadherin 1 on the tumor cells infected with RCASBP*-Bcl-xL* by immunostaining assays, using the two anti–cadherin 1 antibodies (data not shown), raising questions about the specificity of the antibodies used in the assays. Taken together, overexpression of Bcl-xL reduces the levels of cadherin 1 RNA and protein, and loss of cadherin 1 is likely to contribute to cell migration and invasion. However, suppression of cadherin 1 function by dnE-cad is not as efficient as Bcl-xL in promoting invasion of β-cell tumor cells (Figure 8B).

### Bcl-xL Interacts with Myosins

The assembly and organization of the actin cytoskeleton can be regulated by small guanosine triphosphatases (GTPases) [32]. We evaluated whether the activities of four members of the small GTPase family (RHO, CDC42, RAC1, and RAS) were changed in the tumor cells infected with RCASBP*-Bcl-xL,* by measuring the signaling activities of these four small GTPases as reflected in the binding of GTP. Their specific effector proteins were expressed as glutathione S-transferase (GST)–fusion proteins to pull down GTP-bound GTPases, which were detected by Western blotting using antibodies against specific GTPases. However, no significant changes in the signaling strength of RHO, CDC42, RAC1, or RAS were detected in the tumor cells infected with RCASBP*-Bcl-xL,* compared with the uninfected parental cells (data not shown), indicating that altered activities of these four proteins were not responsible for the remodeling of the actin skeleton in the tumor cells infected with RCASBP*-Bcl-xL*. We cannot exclude the possibilities that small GTPase activities vary spatially within the cells or that other members of the family are responsible for the reorganization of the actin cytoskeleton when Bcl-xL is overexpressed.

**Figure 9:**
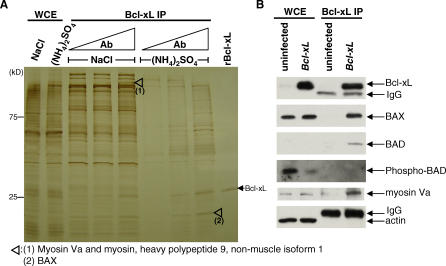
Bcl-xL Interacts with BAX, Unphosphorylated BAD, and Myosin Va in the Tumor Cells Infected with RCASBP*-Bcl-xL* (A) Whole cell extracts of tumor cells infected with RCASBP*-Bcl-xL* were prepared in cell lysis buffer containing 150 mM NaCl or 100 mM (NH_4_)_2_SO_4_ (L100 buffer), and subjected to IP with increasing amounts of an anti-Bcl-xL antibody. The precipitates and 5 ng of recombinant human Bcl-xL (rBcl-xL) were analyzed by SDS-PAGE and silver staining. The putative Bcl-xL-interacting bands (open arrowheads) were excised from the gel for mass spectrometry. The identities of the proteins are listed. Both myosin Va and myosin, heavy polypeptide 9, non-muscle isoform 1 were identified by mass spectrometry using proteins from the excised area labeled “(1).” (B) Whole cell extracts of uninfected tumor cells and tumor cells infected with RCASBP*-Bcl-xL* were prepared in L100 buffer, and subjected to IP with an anti-Bcl-xL antibody. The precipitates were analyzed by SDS-PAGE and Western blotting using specific antibodies against Bcl-xL, BAX, BAD, phospho-BAD, myosin Va, and actin. Four of these proteins, Bcl-xL, BAX, BAD, and myosin Va, were found in anti-Bcl-xL IP from cells infected with RCASBP*-Bcl-xL*.

To further investigate the mechanism by which Bcl-xL increases cell motility, we sought to identify Bcl-xL-interacting proteins by mass spectrometry. Bcl-xL is located both in the cytosol and in mitochondrial membranes [33,34], whereas BCL2, a related anti-apoptotic protein, is exclusively membrane-bound [35]. We postulated that the cytosolic fraction of Bcl-xL might contribute to its effects on cell shape, motility, and invasiveness, so we prepared whole cell extracts from tumor cells infected with RCASBP*-Bcl-xL* in two buffer conditions, and used an anti-Bcl-xL antibody to co-precipitate interacting proteins. The antibody-bound complexes were eluted from beads by boiling, separated by sodium dodecyl sulfate polyacrylamide gel electrophoresis (SDS-PAGE), and visualized by silver staining. Although many protein bands were present in the IP, we observed that the intensities of several protein bands, including Bcl-xL itself, were enhanced with increasing amounts of an anti-Bcl-xL antibody (Figure 9A). These putative Bcl-xL-interacting bands were excised and subjected to tryptic digestion, and peptides were analyzed by mass spectrometry. The identities of the proteins were BAX, myosin Va, and myosin, heavy polypeptide 9, non-muscle isoform 1.

The current view of apoptotic regulation is that Bcl-xL and its relatives bind to and thereby inhibit the pro-apoptotic activity of BAX and BAK1, which are otherwise poised to initiate the apoptotic cell death cascade [36,37]. Activated BH3 sensors, such as BAD, bind to Bcl-xL-related proteins, relieving suppression of BAX and BAK1. We confirmed the interaction between BAX and Bcl-xL in the β-cell tumor cells infected with RCASBP*-Bcl-xL* by IP-Western blotting (Figure 9B). We also observed an interaction between Bcl-xL and BAD, but not phosphorylated BAD (Figure 9B). The significance of the reduction of phosphorylated BAD in the tumor cells infected with RCASBP*-Bcl-xL* remains to be determined.

We obtained an antibody against myosin Va and confirmed the presence of myosin Va in the anti-Bcl-xL IP (Figure 9B), suggesting that the interaction between Bcl-xL and myosin Va was specific and significant. No suitable antibodies against the other implicated myosin were found, so Western blotting to verify its specificity by IP from the cells infected with RCASBP*-Bcl-xL* was not possible. Furthermore, the association between Bcl-xL and myosin Va was unlikely to be mediated by the actin cytoskeleton, because actin was not present in Bcl-xL IP (Figure 9B). Myosins have been implicated in cell movement and migration [38–40], and therefore it is possible that Bcl-xL promotes cell motility through its interaction with myosins.

## Discussion

The studies presented here demonstrate a system to transfer cDNAs into premalignant lesions in mice to analyze their effects on tumor progression. This system utilizes RCASBP avian retroviral vectors to deliver cDNAs into pancreatic β cells in *RIP-Tag; RIP-tva* bitransgenic mice during tumorigenesis. This approach has the advantage of introducing somatic genetic changes in a tissue-specific and time-controlled fashion, which more faithfully mimics sporadic tumor development. It also avoids any potential perturbation of normal tissue formation due to the ectopic expression of the gene of interest during development. It is much faster to generate vectors carrying genes of interest than to generate transgenic mice. In addition, using cell lines derived from β-cell tumors in *RIP-Tag; RIP-tva* bitransgenic mice, further biochemical and phenotypic analysis can be performed, taking advantage of the high efficiency of viral infection in vitro.

The low efficiency of in vivo infection has been a limitation of using the RCAS-TVA system [13,14]. Here, we were able to achieve 10%–20% in vivo infection efficiency in hyperplastic pancreatic islet β-cell lesions using high viral titer (>10^8^ infectious units per milliliter) and the intra-cardiac route to deliver the RCASBP viruses into mice. Since not all the premalignant lesions are infected with the RCASBP vectors and 2%–4% of islets develop into tumors in the *RIP-Tag; RIP-tva* bitransgenic mice without viral infection, only the factors that confer a selective advantage over the natural course of tumorigenesis in *RIP-Tag; RIP-tva* mice can be identified. Moreover, the percentage of RCASBP*-Bcl-xL*-positive tumors decreases over time as the uninfected *RIP-Tag; RIP-tva* cells gradually develop into tumors. In particular, RCASBP vectors carrying genes that promote metastasis will be most easily recognized, because metastasis to pancreatic lymph nodes or other organs does not normally occur in *RIP-Tag; RIP-tva* mice. Although tumors are likely to be clonally expanded from single cells infected with RCASBP vectors carrying progression factors, we were not able to show whether the tumors were clonal because of the limited DNA materials isolated from the small tumors in this study.

We delivered RCASBP viruses carrying tumor progression factors into *RIP-Tag; RIP-tva* bitransgenic animals at 7 wk of age, when hyperplastic islets develop. Analogous to the results obtained from *RIP1-Tag2; RIP1-dnE-cad* bitransgenic mice [22], we demonstrated that introduction of RCASBP*-dnE-cad* into the hyperplastic islets of *RIP-Tag; RIP-tva* animals promoted invasive tumor formation and metastases to the pancreatic lymph nodes. Mice at other ages could also be used to study whether the time at which the candidate factors are introduced influences the outcome, as long as islet cells undergo cell division to allow infection. For example, the transforming growth factor β1 (TGFB1) receptor can act as a tumor suppressor at early stages of tumor development, but at later stages TGFB1 responsiveness promotes invasion and metastasis [41,42]. Moreover, a combination of several RCASBP viruses encoding different factors of interest could be used for infection simultaneously or sequentially, and the proviral DNA could be isolated from especially metastatic malignant tumors to identify single factors or combinations of factors that may contribute to the malignancy. However, this approach is limited by the inability of the RCASBP vector to accommodate cDNAs greater than 2.5 kb in size.

The effects of overproducing Bcl-xL via somatic gene transfer were provocative and unexpected. First, no significant protection against apoptosis was found either in the tumors from mice infected with RCASBP*-Bcl-xL* or in the tumor cells infected with RCASBP*-Bcl-xL* in vitro. These findings are quite distinct from the demonstrable anti-apoptotic effect seen when Bcl-xL was expressed via a transgene in all islet β cells throughout tumorigenic ontogeny in the *RIP1-Tag2* model [25], as well as in MYC transgenic mice [26]. It remains possible that Bcl-xL contributes to cell survival at earlier stages (hyperplasia/angiogenesis) or a later stage (metastasis) in mice infected with RCASBP-*Bcl-xL*. Additionally, we cannot exclude episodic effects on apoptosis when tumors were forming, as opposed to chronic effects detectable at the later time points of our analysis. This apparent insensitivity of β-cell tumors to the anti-apoptotic effect of Bcl-xL deserves future investigation. Notably, a similar phenotype of increased invasiveness without suppression of apoptosis has been observed when another apoptotic modulator was overexpressed in the *RIP1-Tag2* mice. Exogenous production of IGF1R for the IGF1/2 survival factors in all islet β cells caused increased invasiveness and lymph node metastasis, with increased (not decreased) apoptosis in the premalignant stages, while having no impact on the tumor stages [23]. Yet elimination of the gene encoding IGF2 produced highly apoptotic tumors with reduced growth, demonstrating the importance of anti-apoptotic signaling for islet tumorigenesis [43]. The similar consequences of ectopically expressing Bcl-xL and IGF1R may imply mechanistic similarity that should be further explored.

Second, we observed that the actin cytoskeleton was rearranged in invasive tumors infected with RCASBP*-Bcl-xL,* presumably contributing to increased motility. In addition, tumor cells infected with RCASBP*-Bcl-xL* in vitro displayed altered morphology, abnormal cortical distribution of the actin cytoskeleton, and elongated cell shape. The morphological changes in tumor cells infected with RCASBP*-Bcl-xL* may contribute to enhanced cell migration and invasion as observed in the two-chamber assays.

Third, as measured by Western blotting and cDNA microarray expression profiling, the epithelial cell–cell junction protein, cadherin 1, was down-regulated and a transcriptional repressor of cadherin 1, snail homolog 1, was up-regulated in the tumor cells infected with RCASBP*-Bcl-xL*. These changes may contribute to the rearrangement of the actin cytoskeleton and diminish the strength of cellular adhesion. Indeed, tumor cells infected with RCASBP*-Bcl-xL* exhibited a strong capability to migrate through transwell membrane and to invade through Matrigel, but the mechanism by which Bcl-xL regulates cadherin 1 remains to be determined.

Finally, the identification of a novel Bcl-xL-interacting protein, myosin Va, may help to explain the pro-invasive activity of Bcl-xL. Myosins belong to a superfamily of actin-based motor proteins comprising at least 15 classes [39]. There are two main groups of myosins: the conventional, well-characterized myosin II class of muscle and non-muscle cells and the unconventional myosins. The myosin II class is referred to as “conventional myosin” because for many years this was the only class of myosin known; members of this class are able to form filaments and are involved in muscle contraction, cell migration, and cytokinesis. The functions of most of the unconventional myosins are not known, but some have been shown to participate in the extension of processes at the leading edge of crawling cells. For example, myosin I is required for formation of pseudopod extensions in the amoeba *Dictyostelium,* and myosin V for extension of filopodia in neurons [44,45].

By comparing the effects of suppressing the function of cadherin 1 with dnE-cad and overproducing Bcl-xL, we have highlighted the provocative pro-invasive activity of Bcl-xL. Cadherin 1 is a well-established barrier to invasive growth in many other epithelial cancers [46], and its suppression enables invasive growth of pancreatic β-cell tumors in transgenic mice [22]. Yet Bcl-xL produced a more highly invasive phenotype in a Matrigel invasion assay of cultured tumor cells than did dnE-cad, supporting the proposition that Bcl-xL has roles other than suppression of cadherin 1 that enhance the invasive growth phenotype. The levels of endogenous cadherin 1 in these two types of cells could contribute to the differences. Whereas endogenous cadherin 1 protein levels were not affected when the dominant-negative form was introduced into the cells, cadherin 1 was down-regulated in the tumor cells infected with RCASBP*-Bcl-xL*. This effect could reduce cell–cell adhesion and also affect the organization of the actin cytoskeleton. On the other hand, the frequency of lymph node metastasis was similar in *RIP-Tag; RIP-tva* mice infected with RCASBP*-Bcl-xL* and RCASBP*-dnE-cad*. It is possible that the β-cell tumor microenvironment in vivo is less discriminating than Matrigel, a solubulized basement membrane preparation extracted from a mouse sarcoma [47].

The BCL2 family proteins function in carcinogenesis by preventing apoptosis of tumor cells, instead of promoting cell proliferation [48]. Of the BCL2 family members, BCL2 and Bcl-xL are most closely related to each other, and repress cell death through common mechanisms [49,50]. However, several lines of evidence indicate that they are not functionally equivalent in tumorigenesis. In primary breast cancer, overexpression of *Bcl-xL* is associated with higher tumor grade and nodal metastasis, and overexpression of *Bcl2* is correlated with lower tumor grade and smaller tumor size [51]. Moreover, nude mice with orthotopic implants of human breast cancer cells transfected with Bcl-xL, but not BCL2, develop lymph node metastasis [52]. Our findings suggest that Bcl-xL enhances cell motility, remodels the actin cytoskeleton, down-regulates cadherin 1, and interacts with myosin Va without affecting cell proliferation or apoptosis. It remains to be investigated whether these properties are unique to Bcl-xL among the BCL2 family members.

In conclusion, we have developed a mouse model to assess the effect of candidate genes in tumor progression, without the need to generate conditional transgenic lines in which genes of interest are expressed at different stages of tumorigenesis. Thus far, we have employed this approach to improve our knowledge of the contribution of Bcl-xL to tumor invasion and metastasis. We postulate that Bcl-xL has a pro-invasive function other than its anti-apoptotic activity. Given that many somatic gene mutations and altered expression of thousands of genes have been discovered in cancers, it will not be a simple task to verify that each candidate gene is important during carcinogenesis. By delivering libraries of retroviruses encoding candidate factors or inhibitory RNAs into transgenic mice, it may be possible to screen many of the genes for effects during tumorigenesis in a variety of tissues.

## Materials and Methods

### Mouse strains.

The elastase-*tva* transgene construct [53] was digested with BamHI and HindIII to release a 800-bp *tva* cDNA fragment encoding the glycosylphosphatidylinisotol-anchored form of the receptor. This cDNA fragment was cloned into a BglII- and HindIII-digested pSG5 vector with an expanded multiple cloning site (gift of E. Emison, Johns Hopkins University). The *tva* cDNA was then released by XbaI/HindIII digestion of the pSG5-*tva* plasmid and subcloned into a XbaI/HindIII-digested RIP-DIPA plasmid that contains the RIP 5′ to the XbaI site and the SV40 small t intron and terminator sequences 3′ to the HindIII site. The resulting *RIP-tva* transgene construct was released from the pBR322 vector backbone by digestion with BamHI, purified, and resuspended in TE for pronuclear injection. *RIP-tva* transgenic founders on a CBA/CAJ × C57BL/6 mixed genetic background were backcrossed for ten generations onto a pure C57BL/6 background. The *RIP-tva* mice were crossed with the previously described *RIP1-Tag2* mice [11] to obtain *RIP-Tag; RIP-tva* mice. All mice were housed in accordance with institutional guidelines. Genotypes were determined by PCR using tail DNA. PCR primers for TVA were 5′-GCCCTGGGGAAGGTCCTGCCC-3′ and 5′- CTGCTGCCCGGTAACGTGACCGG-3′.

### β-cell tumor cell line.

A pancreatic β-cell tumor cell line (βTC-N134) was derived from a tumor from a *RIP-Tag; RIP-tva* bitransgenic animal at 16 wk of age, and maintained in Dulbecco's Modified Eagle Medium (DMEM) supplemented with 10% fetal bovine serum (FBS), 0.2 mM L-glutamine, and 1% penicillin/streptomycin in a humidified 37 °C incubator under 5% CO_2_; method modified from [54].

### Viral propagation and delivery.

RCASBP is a replication-competent avian leukosis virus with a splice acceptor and the Bryan-RSV *pol* gene. RCASBP*-ALPP* has been described previously [55]. RCASBP*-GFP* was a gift from Maureen Peters and Constance Cepko, Harvard Medical School. Human *Bcl-xL* cDNA was a gift from Stanley Korsmeyer, Dana-Farber Cancer Institute. RCASBP-*dnE-cad* (myc-tagged, mouse origin) and RCASBP*-Bcl-xL* were generated by Yi Li and William Pao, respectively, in the Varmus laboratory. The production of the dnE-cad and Bcl-xL was verified by Western blot analysis. Chicken fibroblasts DF-1 [56,57] transfected with RCASBP vectors were maintained in DMEM supplemented with 10% FBS, 0.2 mM L-glutamine, and 1% penicillin/streptomycin in a humidified 37 °C incubator under 5% CO_2_. For in vitro infection, RCASBP viral supernatant was passed through a 0.45-μm filter to obtain cell-free viruses and used as growth medium for the *RIP-Tag; RIP-tva* pancreatic β-cell tumor cell line once a day for 2–3 d. For in vivo infection, viral supernatant was passed through 0.45-μm filters and was concentrated by high-speed ultracentrifugation at 23,000 rpm for 1.5 h before intra-cardiac injection into mice. Intra-cardiac injection was performed as described [58]. Viral titer was determined by end-point dilution of DF-1 producer cells. PCR primers for RCASBP were 5′-ACCGGGGGATGCGTAGGCTTCA-3′ and 5′-CCGCAACACCCACTGGCATTACC-3′.

### Tissue preparation.

Tissues were removed and either fixed in 10% buffered formalin overnight at room temperature or immediately placed in OCT and frozen on dry ice. Fixed and frozen tissues were processed and cut into 5-μm and 10-μm sections, respectively, at Histoserv (http://www.histoservinc.com/index.php).

### Immunohistochemical analysis.

Formalin-fixed/paraffin-embedded sections were deparaffinized and rehydrated by passage through a graded xylene/ethanol series before staining. Cells were cultured on glass chamber slides for 3 d before fixation in 4% paraformaldehyde in PBS for 20 min, and were permeabilized by 0.1% Triton X-100 (in PBS) for 4 min. Immunochemistry with VECTASTAIN Elite ABC Kit (Vector Laboratories, http://www.vectorlabs.com/) was performed according to the manufacturer's instructions. Primary antibodies used were mouse anti–SV40 T antigen (1:400; Calbiochem, http://www.emdbiosciences.com/html/CBC/home.html), rabbit anti-insulin (1:600; Immunostar, http://www.immunostar.com/), rabbit anti-synaptophysin (1:100; Dako, http://www.dako.com/), rabbit anti-Ki67 (1:1,000; Novocastra, Vision BioSystems, http://www.leica-microsystems.com/biosystems.html), and rabbit anti–activated caspase 3 (1:100; Cell Signaling Technology, http://www.cellsignal.com/).

### Immunofluorescent analysis.

Frozen sections were air-dried overnight at room temperature and fixed in cold acetone for 10 min. Formalin-fixed/paraffin-embedded sections were deparaffinized and rehydrated by passage through a graded xylene/ethanol series. Cells were cultured on glass chamber slides for 3 d before fixation in 4% paraformaldehyde in PBS for 20 min, and permeabilized by 0.1% Triton X-100 (in PBS) for 4 min. After blocking, slides were incubated with primary antibodies overnight at 4 °C or for 1 h at room temperature. After three washes of TBS-T, slides were incubated with secondary antibodies for 1 h at room temperature. Primary antibodies used were rabbit anti-TVA (1:200; gift of Andrew Leavitt, University of California San Francisco), goat anti-mouse PDX1 (1:2,000; gift of Chris Wright, Vanderbilt University), and rhodamine-phalloidin (1:40; Invitrogen, http://www.invitrogen.com/). TRITC-donkey anti-rabbit IgG (1:200) and FITC-donkey anti-goat IgG (1:200) were purchased from Jackson ImmunoResearch Laboratories (http://www.jacksonimmuno.com/). To stain DNA, slides were incubated with DAPI (4′,6-diamidino-2-phenylindole; 5 μg/ml in PBS) for 15 min at room temperature.

### FACS.

Pancreases were perfused and digested into small pieces with collagenase (Sigma-Aldrich, http://www.sigmaaldrich.com/) at 37 °C for 15 min, and were centrifuged in a Ficoll gradient (11%, 20.5%, 23%, and 25% [w/v]) as described [19]. Partially digested pancreatic tissues in the top two layers of the gradient were further digested and dispersed into single-cell suspension as described [20]. After blocking with 3% donkey serum at 4 °C for 30 min, cells were incubated with rabbit anti-TVA antibodies at 4 °C for 30 min, incubated with donkey Cy5-conjugated anti-rabbit antibodies at 4 °C for 30 min, and analyzed for Cy5 and GFP by FACS using a BD Biosciences (http://www.bdbiosciences.com/) FACS-DiVa Cell Sorter or FACSCalibur flow cytometer.

### Western blot analysis.

To prepare whole cell extracts, cells were lysed in buffer containing 100 mM NaCl, 100 mM Tris (pH 8.2), 0.5% NP-40, and protein inhibitor cocktail at 4 °C. Then 20 μg of total proteins were separated by SDS-PAGE and electrotransferred onto nitrocellulose membranes. The membranes were stained with Ponceau S and immunoblotted with rabbit anti-Bcl-xL antibody (1:1,000; Cell Signaling Technology), mouse anti–cadherin 1 antibody (clone 36, 1:1,000; BD Biosciences), rabbit anti-actin antibody (1:5,000; Sigma-Aldrich), rabbit anti–snail homolog 1 (1: 1,000; Abcam, http://www.abcam.com/), rabbit anti-BAX antibody (1:1,000; Cell Signaling Technology), rabbit anti-BAD antibody (1:1,000; Cell Signaling Technology), mouse anti-phospho-BAD (Ser112) antibody (1:1,000; Cell Signaling Technology), rabbit anti–myosin Va antibody (1:1,000; Sigma-Aldrich), and appropriate secondary antibodies. The proteins were detected with ECL chemiluminescent substrate (GE Healthcare, http://www.gehealthcare.com/) on Kodak (http://www.kodak.com/) BioMax MR films.

### In vitro migration and invasion assays.

Transwell chambers with 8-μm porous polycarbonate membranes (Corning, http://www.corning.com/) and invasion chambers with 8-μm porous polyester membranes and coated with Matrigel basement membrane matrix (BD Biosciences) were used. One million cells were plated in the upper chambers in DMEM containing 2% FBS, 0.2 mM L-glutamine, and 1% penicillin/streptomycin. The lower chambers were filled with DMEM containing 10% FBS, 0.2 mM L-glutamine, and 1% penicillin/streptomycin. After a 72-h incubation in a humidified 37 °C incubator under 5% CO_2_, cells migrating or invading into the bottom chambers were fixed, stained with hematoxylin, and counted in five fields under 200× magnification.

### IP.

To prepare whole cell extracts for anti-Bcl-xL IP, cells were lysed in cell lysis buffer containing 150 mM NaCl according to the manufacturer's instructions from Cell Signaling Technology or in L100 buffer [59] containing protein inhibitor cocktail at 4°C for 30 min. The extracts were precleared with protein A Sepharose beads for 1 h at 4 °C. Rabbit anti-Bcl-xL antibody (Cell Signaling Technology) was mixed with the extracts for at least 4 h at 4 °C, and then protein A Sepharose beads were added for 1 h at 4 °C. The precipitates were washed extensively and were eluted from the beads by boiling in Laemmli buffer. Recombinant human Bcl-xL and BCL2 proteins were purchased from R&D Systems (http://www.rndsystems.com/). Anti–cadherin 1 IP using two anti–cadherin 1 antibodies—clone ECCD-2 (Zymed, http://www.invitrogen.com) and clone 36 (BD Biosciences)—was performed as described [60] except that protein A/G plus-agarose (Santa Cruz Biotechnology, http://www.scbt.com/) was used instead of protein A Sepharose beads.

## Supporting Information

### Accession Numbers

The Entrez Gene (http://www.ncbi.nlm.nih.gov/sites/entrez?db=gene) accession numbers for gene products discussed in this paper are BAD (12015), BAX (12028), cadherin 1 (12550), myosin Va (17918), myosin, heavy polypeptide 9, non-muscle isoform 1 (17886), PDX1 (18609), snail homolog 1 (20613), and TVA (420066).

## Abbreviations

ALPP: human placental alkaline phosphatase
DMEM: Dulbecco's Modified Eagle Medium
dnE-cad: a dominant-negative form of cadherin 1
F-actin: filamentous actin
FACS: fluorescence-activated cell sorting
FBS: fetal bovine serum
GFP: green fluorescent protein
GTPase: guanosine triphosphatase
IC-1: invasive carcinoma type 1
IC-2: invasive carcinoma type 2
IGF: insulin-like growth factor
IP: immunoprecipitation
IT: noninvasive islet tumor
RCASBP: subgroup A replication-competent avian leukosis virus with a splice acceptor and the Bryan-RSV *pol* gene
RIP: rat insulin promoter
SDS-PAGE: sodium dodecyl sulfate polyacrylamide gel electrophoresis
